# Tocilizumab Improves the Prognosis of COVID-19 in Patients with High IL-6

**DOI:** 10.3390/jcm10081583

**Published:** 2021-04-09

**Authors:** Robert Flisiak, Jerzy Jaroszewicz, Magdalena Rogalska, Tadeusz Łapiński, Aleksandra Berkan-Kawińska, Beata Bolewska, Magdalena Tudrujek-Zdunek, Dorota Kozielewicz, Marta Rorat, Piotr Leszczyński, Krzysztof Kłos, Justyna Kowalska, Paweł Pabjan, Anna Piekarska, Iwona Mozer-Lisewska, Krzysztof Tomasiewicz, Małgorzata Pawłowska, Krzysztof Simon, Joanna Polanska, Dorota Zarębska-Michaluk

**Affiliations:** 1 Department of Infectious Diseases and Hepatology, Medical University of Białystok, 15-089 Białystok, Poland; pmagdar@gmail.com (M.R.); twlapinski@gmail.com (T.Ł.); 2 Department of Infectious Diseases and Hepatology, Medical University of Silesia, 40-055 Katowice, Poland; jerzy.jr@gmail.com; 3 Department of Infectious Diseases and Hepatology, Medical University of Łódź, 90-549 Łódź, Poland; aleksandra.berkan@gmail.com (A.B.-K.); annapiekar@gmail.com (A.P.); 4 Department of Infectious Diseases, University of Medical Sciences, 61-701 Poznań, Poland; bbolewska@ump.edu.pl (B.B.); iwonalisewska@poczta.onet.pl (I.M.-L.); 5 Department of Infectious Diseases and Hepatology, Medical University of Lublin, 20-059 Lublin, Poland; magdalena.tudrujek@gmail.com (M.T.-Z.); tomaskdr@poczta.fm (K.T.); 6 Department of Infectious Diseases and Hepatology, Faculty of Medicine, Collegium Medicum in Bydgoszcz, Nicolaus Copernicus University, 87-100 Toruń, Poland; d.kozielewicz@wsoz.pl (D.K.); mpawlowska@cm.umk.pl (M.P.); 7 Department of Forensic Medicine, Wrocław Medical University, 50-367 Wrocław, Poland; marta.rorat@gmail.com; 8 First Infectious Diseases Ward, Gromkowski Regional Specialist Hospital in Wrocław, 51-149 Wrocław, Poland; 9 Department of Rheumatology, Rehabilitation and Internal Medicine, Poznan University of Medical Sciences, 61-701 Poznań, Poland; piotr_leszczynski@wp.pl; 10 Department of Rheumatology and Osteoporosis, Szpital im. J. Strusia w Poznaniu, Szpital im. J. Strusia, 61-285 Poznań, Poland; 11 Department of Infectious Diseases and Allergology, Military Institute of Medicine, 04-141 Warsaw, Poland; kklos@wim.mil.pl; 12 Department of Adults’ Infectious Diseases, Medical University of Warsaw, 02-091 Warsaw, Poland; jdkowalska@gmail.com; 13 Department of Infectious Diseases, Jan Kochanowski University, 25-369 Kielce, Poland; pabjan3@tlen.pl (P.P.); dorota1010@tlen.pl (D.Z.-M.); 14 Department of Infectious Diseases and Hepatology, Wrocław Medical University, 50-367 Wrocław, Poland; krzysimon@gmail.com; 15 Department of Data Science and Engineering, Silesian University of Technology, 44-100 Gliwice, Poland; Joanna.Polanska@polsl.pl

**Keywords:** COVID-19, SARS-CoV-2, interleukin-6, tocilizumab, therapy

## Abstract

Despite direct viral effect, the pathogenesis of coronavirus disease 2019 (COVID-19) includes an overproduction of cytokines including interleukin 6 (IL-6). Therefore, tocilizumab (TOC), a monoclonal antibody against IL-6 receptors, was considered as a possible therapeutic option. Patients were selected from the SARSTer database, containing 2332 individuals with COVID-19. Current study included 825 adult patients with moderate to severe course. Analysis was performed in 170 patients treated with TOC and 655 with an alternative medication. The end-points of treatment effectiveness were death rate, need for mechanical ventilation, and clinical improvement. Patients treated with TOC were balanced compared to non-TOC regarding gender, age, BMI, and prevalence of coexisting conditions. Significant effect of TOC on death was demonstrated in patients with baseline IL-6 > 100 pg/mL (hazard ratio [HR]: 0.21, 95% confidence interval [CI]: 0.08–0.57). The best effectiveness of TOC was achieved in patients with a combination of baseline IL-6 > 100 pg/mL and either SpO2 ≤ 90% (HR: 0.07) or requiring oxygen supplementation (HR: 0.18). Tocilizumab administration in COVID-19 reduces mortality and speeds up clinical improvement in patients with a baseline concentration of IL-6 > 100 pg/mL, particularly if they need oxygen supplementation owing to the lower value of SpO2 ≤ 90%.

## 1. Introduction

A novel coronavirus named severe acute respiratory syndrome coronavirus 2 (SARS-CoV-2) was identified in December 2019 and found to be responsible for an outbreak of respiratory tract infections discovered in Wuhan, China. The outbreak of the disease known as a coronavirus disease 2019 (COVID-19) was announced as a global pandemic by the World Health Organization (WHO) in March 2020. The search for effective therapy focused on repurposing of approved drugs with confirmed activity against other viruses, that included, for example, remdesivir (RDV), which was previously studied for the treatment of Ebola virus disease as well as SARS-CoV-1 and middle east respiratory syndrome (MERS) coronaviruses [1,2]. Based on findings from phase III clinical trials and real-world experience study, RDV received both American and European authorization [3,4,5]. Recommendations were also given to low-molecular-weight heparin and dexamethasone [6,7]. However, the pathogenesis of COVID-19 is complicated and includes, in addition to direct viral effect and coagulopathy, an overproduction of proinflammatory cytokines termed a cytokine storm, which is responsible for organ damage and is considered a major reason for death due to COVID-19 [8]. Unfortunately, standard anti-inflammatory treatments appear to be insufficient for controlling the cytokine storm. Concentrations of several proinflammatory cytokines, including interleukin (IL)-6, are substantially increased in patients with severe COVID-19 [9]. Higher concentrations of IL-6 was shown to be associated with faster progression of the disease and worse prognosis. Therefore, tocilizumab (TOC), an inhibitor of the IL-6 receptors, was considered as a possible therapeutic option [9,10,11]. Data from several studies have been contradictory mostly because of the difficulties in the selection of optimal population and finding the proper stage of the disease for administration [12,13,14]. Although the most recent randomized, double-blind, placebo-controlled trial by Stone et al. was not able to confirm the effectiveness of TOC, authors did not exclude the possible benefit from interleukin-6 receptor blockade in some patient populations because of wide confidence intervals for efficacy comparisons [14].

The purpose of the study is to search for the population of patients with severe COVID-19, which could obtain maximal benefit from the administration of tocilizumab, and identify the predictors of response to the treatment with this drug.

## 2. Materials and Methods

Patients were selected from the SARSTer national database, which included 2332 patients treated between 1 March and 31 October 2020 in 30 Polish centers. This ongoing project, supported by the Polish Association of Epidemiologists and Infectiologists, is a national real-world experience study assessing treatment in patients with COVID-19. The decision about the treatment regimen was taken entirely by the treating physician concerning current knowledge and recommendations of the Polish Association of Epidemiologists and Infectiologists [15,16,17]. The SARSTer study was approved by the Ethical Committee of the Medical University of Białystok. If necessary, the local bioethics committees approved experimental use of drugs in patients with COVID-19. Patients aged below 18 years, those with oxygen saturation >95%, or acute respiratory distress syndrome (ARDS) at baseline were excluded from the database of 2332 patients. As a result, the current study included 825 adult patients with moderate to severe course of the disease.

Among those 825 patients, the retrospective analysis was carried out in 170 patients treated with tocilizumab (RoActemra, Roche Pharma AG) and 655 patients who did not receive this medication as well as any other monoclonal antibody directed against cytokine receptors. Tocilizumab was administered intravenously at 8 mg/kg (maximum dose: 800 mg) in a single dose (1-h infusion) after exclusion of severe bacterial and HBV infection. If no improvement was observed, the second dose was considered after 8 to 12 h (administered in 42% patients) according to the national recommendations [15,16,17]. Data were entered retrospectively and submitted online by a web-based platform operated by Tiba sp. z o.o. Parameters collected at baseline included age, gender, body mass index (BMI), coexisting conditions, other medication-related to COVID-19, clinical status at admission, and adverse events. Baseline clinical status at hospital admission was classified according to oxygen saturation (SpO2) 91–95%, or SpO2 ≤ 90%, as well as based on the score on an ordinal scale.

The end-points of treatment effectiveness were rate of death, need for mechanical ventilation, and clinical improvement in the ordinal scale based on WHO recommendations modified to fit the specificity of the national health care system. Clinical improvement was defined as at least a 2-point decrease from baseline to 14, 21, and 28 days of hospitalization. The ordinal scale was scored as follows: (1) unhospitalized, no activity restrictions; (2) unhospitalized, no activity restrictions and/or requiring oxygen supplementation at home; (3) hospitalized, does not require oxygen supplementation and does not require medical care; (4) hospitalized, requiring no oxygen supplementation, but requiring medical care; (5) hospitalized, requiring normal oxygen supplementation; (6) hospitalized, on non-invasive ventilation with high-flow oxygen equipment; (7) hospitalized, for invasive mechanical ventilation or extracorporeal membrane oxygenation (ECMO); (8) death.

To identify possible predictors of response to the treatment with TOC, we compared rates of achieved end-points in patients receiving versus not receiving TOC. The following baseline predictors were included: age above 70 years, the need for oxygen high flow (ordinal scale 6 points) at baseline, clinical worsening during 7 days of hospitalization in patients with regular oxygen supplementation at baseline (5 points in original scale), SpO2 < 90% at baseline, and several laboratory measures at baseline, such as IL-6 > 100 pg/mL, C-reactive protein (CRP) > 200 mg/L, neutrophils > 7500/µL, lymphocytes > 1200/µL, D-dimer > 1000 µg/L, and procalcitonin > 0.1 ng/mL.

### Statistical Analysis

The results are expressed as mean ± standard deviation (SD) or n (%). *p* values of <0.05 were considered to be statistically significant. The significance of difference was calculated by Fisher’s exact test for nominal variables and by Mann–Whitney U and Kruskal Wallis ANOVA for continuous and ordinal variables. Due to the highly variable group size, the Fisher’s *p*-values were accompanied by odds ratio (OR) as the effect size measure independent of the sample size. The association between variables was measured by Spearman’s rank correlation coefficient and its significance test *p*-values. Survival analyses were performed by log-rank (Mantel–Cox) test supported by the Mantel–Haenszel hazard ratio (MH HR) and its 95% confidence interval as the effect size measure and depicted as Kaplan–Meier (KM) plots. The threshold value of IL-6, splitting between the low and the high IL-6 level groups, was found as maximizing the non-TOC vs. TOC hazard ratio value in the high IL-6 group providing the significant differences between the IL-6 group-specific KM survival functions (as measured by log-rank test *p*-value). Univariable comparisons were calculated by GraphPad Prism 5.1 (GraphPad Software, Inc., La Jolla, CA, USA).

## 3. Results

Among 825 patients included in the study, 170 received therapy with TOC and 655 did not receive TOC. As shown in Table 1, groups were balanced based on gender, age, and BMI, but there was a predominance of males in both arms. Patients treated with TOC more frequently demonstrated a course of the disease with SpO2 ≤ 90% at admission to the hospital (65.9%) compared to those without TOC (37.7%). Moreover, patients treated with TOC more often required normal or high flow oxygen supplementation (93.6%) compared to the non-TOC group (76.8%). The prevalence of coexisting conditions was similar in both groups, but patients treated with TOC more frequently received other medications related to COVID-19 (Table 1).

**Figure 1:**
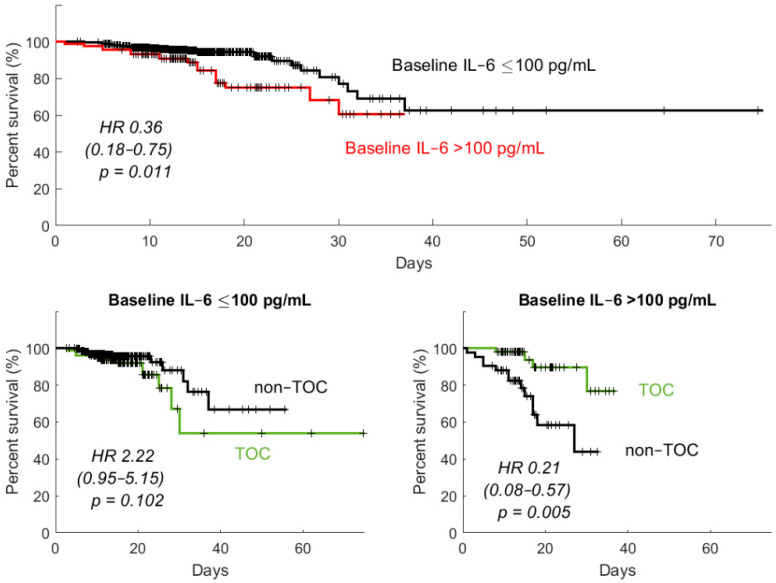
Kaplan–Meier graphs demonstrating the effect of tocilizumab versus no tocilizumab administration on patients’ survival depending on the obtained optimal threshold baseline serum concentration of interleukin 6. The hazard ratios (HR) and their 95% confidence intervals are provided as well as the log-rank test p-values.

As shown in Table 2, the rate of clinical improvement after 21 and 28 days was significantly better in patients who did not receive TOC. However, a statistically significant effect of TOC on rates of death was demonstrated in patients with baseline IL-6 exceeding 100 pg/mL or those needing oxygen supplementation at baseline whose condition worsened within the initial 7 days of hospitalization (Table 2, Figure 1). As shown with the Kaplan–Meier analysis, there were no significant differences between TOC and non-TOC arms when the analysis was carried out in all patients or those with baseline IL-6 concentration below 100 pg/mL. Further analysis included the correlation between IL-6 concentration and several possible clinical and laboratory indices associated with the course of the disease. Among patients with baseline SpO2 ≤ 90%, who are potential TOC recipients, significant correlation was demonstrated among serum concentrations of IL-6 and SpO2, levels of C-reactive protein, procalcitonin D-dimers, as well as white blood cell and neutrophil counts (Table 3).

**Figure 2:**
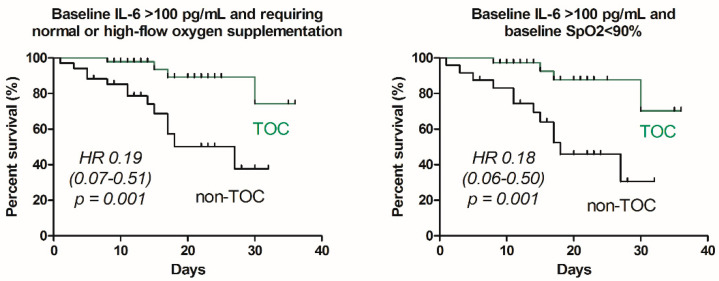
Kaplan–Meier graphs demonstrating the effect of tocilizumab versus no tocilizumab administration on patients’ survival depending on baseline serum concentration of interleukin 6. The hazard ratios (HR) and their 95% confidence intervals are provided as well as the log-rank test *p*-values.

To improve the predictive value, a combination of several measures was also analyzed. As shown in Table 4, the best effectiveness of TOC administration can be achieved in patients with serum IL-6 > 100 pg/mL and either SpO2 ≤ 90% or requiring normal or high-flow oxygen supplementation. Statistically significant effectiveness was achieved regarding the risk of death, the need for mechanical ventilation, as well as clinical improvement after 21 and 28 days (Table 4). Significantly better survival among such patients treated with TOC was also demonstrated with a Kaplan–Meier analysis (Figure 2).

### TOC

Adverse events related to therapy were infrequent and reported in 21.7% and 17.3% of patients in TOC and non-TOC arms, respectively. As shown in Table 5, the most frequent was an elevation of ALT activities, diarrhea, and prolonged QT interval. Prevalence of adverse events was similar in both arms (Table 5). No secondary infections were noticed in patients treated with TOC.

## 4. Discussion

Uncontrolled immune activation with high-level release of various pro-inflammatory cytokines is a hallmark of not only the lung damage but also multiorgan damage during later phases of COVID-19. It is usually termed as a “cytokine storm” and observed mainly during the second and third week of symptomatic disease in patients with severely impaired oxygen saturation [18]. Unsurprisingly, anti-cytokine agents including anti-IL-1R and anti-IL6R antagonists were among important candidates in the therapy of later stages of COVID-19. One of the rationales was a good suppressive effect of tocilizumab in cytokine release syndrome during CAR T-cells therapy [19]. Regardless of the pathophysiological link, various randomized and observational studies of TOC, while suggesting some benefit, did not bring clear evidence supporting its use in COVID-19. This may reflect the unique features of individual immune responses to pathogens, as well as the necessity of a complex and personalized approach in prescribing and timing immunomodulatory treatment. Proposing clinical trial protocol taking such diversity into account proves to be challenging; thus, personalized medicine relies on observational research and real-life experiences.

Results of our real-world evidence study could not only potentially explain the lack of effect of TOC observed in other studies but also provide information on the optimal use of this agent. In our cohort, similar to the first two randomized controlled trials (RCT), TOC did not decrease overall mortality in hospitalized patients with COVID-19. In the first study by Stone JH et al. [14], 83% of 243 COVID-19 subjects requiring oxygen supplementation but not mechanical ventilation were randomized to TOC (8 mg/kg, single dose) or placebo. In this study, the hazard ratio (HR) for death was 0.83, which was not significant, with broad 95% confidence intervals (0.38 to 1.81, *p* = 0.64) suggesting heterogeneity of effect probably depending on other clinical variables not found in the publication. In another RCT by Hermine et al. [12] including 131 patients with COVID-19 pneumonia requiring oxygen supplementation but not mechanically ventilated, 64 were randomized to TOC (8 mg/kg, twice) or placebo. Likewise, the adjusted HR for 28-day mortality was 0.92 (90%CI 0.33–2.53). On the other hand, on day 14 in TOC-group, 12% fewer patients needed non-invasive or mechanical ventilation, or 12% less died (HR 0.58; 90%CI 0.33–1.00). Only the most recent and largest RCT by Salama et al. [13] showed a survival benefit in patients treated with TOC. In this study, 377 subjects with COVID-19 pneumonia, 64% requiring low-flow and 26% on non-invasive and high flow oxygen supplementation, were randomized to TOC (n = 259, 8 mg/kg, one or two doses) or placebo. By day 28, HR for mechanical ventilation or death was 0.56 (95%CI: 0.33–0.97, *p* = 0.04), while still the number of patients who died by that day of any reason was 10.4% in TOC group vs. 8.6% in the placebo group.

Summarizing all aforementioned randomized clinical trials despite a rather homogenous population included, which is patients with COVID-19 pneumonia mainly requiring oxygen supplementation but not ventilated, it was not possible to visualize the obvious survival benefit of TOC. Similarly in our study, the overall mortality was comparable between TOC and non-TOC patients (odds ratio, OR 1.05; 95%CI: 0.61–1.80). Furthermore, the group receiving TOC showed even lesser odds of clinical improvement after 14 and 21 days of therapy (OR 0.61 and 0.64, respectively), while it was comparable to the standard-of-care therapy at the end of observation, i.e., after 28 days (OR 0.87). On the other hand, only analyses in specific subgroups showed not only a survival benefit but also a more rapid clinical improvement in patients treated with TOC. It was quite striking that in previous studies, mortality HR had quite outsized confidence intervals, suggesting another factor playing the predictive role of TOC efficacy. Surprisingly enough, cited above RCT did not evaluate baseline IL-6 serum concentration even when TOC is aimed at blocking an IL-6 proinflammatory pathway. Indeed, we performed a detailed subgroup analysis aiming at the development of predictors of TOC response in COVID-19 subjects. Not unpredictably, the best response to TOC concerning decreasing 28-day mortality (OR = 0.27; 95%CI: 0.10–0.78, 11% vs. 31%, *p* = 0.02) was observed in subjects with baseline serum IL-6 > 100 pg/mL, while it was not observed in subject with baseline IL-6 50–100 pg/mL and below 50 pg/mL. Importantly, an IL-6 level of more than 100 pg/mL observed in approximately 18% of all studied patients possibly explain why the difference was not noted in overall studied groups in the aforementioned RCT but also in our study. This observation also underlines the pathogenetic complexity of cytokine imbalance during COVID-19. It is known that cytokine storm in COVID-19 consists of various, not necessarily overlapping, soluble immune mediators (SIMs) including IL-1β, IL-6, IL-8, and tumor necrosis factor-alpha (TNF-α) which could yield different predictive value [20]. Interestingly, Mathew et al. [21] in their elegant study had shown at least three different immunotypes of COVID-19, 1–3, depending on the cluster of differentiation of (CD)4+ cell, CD8+ cell, and B-cell, and plasmablasts activation/exhaustion, which was associated with different outcomes but also most likely with different levels of cytokines. Interestingly, despite baseline IL-6, the effect of TOC in our study did not depend on baseline CRP, D-dimer, or lymphocyte concentrations, which are also regarded as factors associated with prognosis [22].

Another important finding of our study was that the highest reduction in mortality, the need for mechanical ventilation, and best clinical improvement at day 28 in patients receiving TOC vs. standard-of-care (SOC) therapy was observed in patients with baseline IL-6 > 100 pg/mL and SpO2 < 90% (11 vs. 50%, 5 vs. 29%, and 75 vs. 37%, respectively), which was not the case in subjects with SpO2 ≥ 90%. This observation might further underline that in subjects with severe hypoxia, further deregulation between IL-6 levels and other cytokines is present and possibly IL-6 activation is deeper and not counterbalanced by regulatory mechanism, which could explain why the effect of TOC is more significant. In addition, in our study, correlation analyses showed the correlation pattern of IL-6 and some soluble immune mediators are different in patients with oxygen saturation lower and higher/equal to 90%.

The results of our study should be taken with some caution because of its retrospective real-world evidence design and because of the smaller number of participants in some subgroup analyses. Moreover, some patients in both arms received additional medication, which could affect the outcome of the disease. On the other hand, patients receiving TOC and non-TOC SOC therapies seem to be well balanced with regard to comorbidities and co-medications for COVID-19, and undoubtedly its advantage is the assessment of baseline serum IL-6 concentration. Indeed, while only one other single-center study showed a beneficial effect of TOC mainly in patients with higher IL-6 [23], our data in a real-world large dataset seems to guide the effective use of TOC in COVID-19. Safety profile of TOC in our study was good. Adverse events were infrequent and mild. However, risk of secondary infections should always be considered [24].

## 5. Conclusions

In conclusion, the possible benefit from the treatment of COVID-19 with tocilizumab can be achieved in selected subpopulations only. This regimen can reduce mortality and the need for mechanical ventilation in patients with a baseline concentration of interleukin 6 exceeding 100 pg/mL, particularly if they need oxygen supplementation due to oxygen saturation of ≤90%. Patients who worsened within the initial 7 days of hospitalization can also obtain some benefits from tocilizumab administration, but it should be clarified in further studies on a larger number of patients.

## Figures and Tables

**Table 1:** Baseline demographic and clinical characteristics of included patients.

Characteristic	All Patients *n* = 825	Tocilizumab *n* = 170	No Tocilizumab *n* = 655	*p*
**Age**				
Mean (SD)	63.1 (15.1)	63.2 (13.8)	63.0 (15.4)	0.94
>70 years (%)	267 (32.4)	53 (31.2)	214 (32.7)	0.78
**Gender**				
Female, *n* (%)	337 (40.8)	60 (35.3)	277 (42.3)	0.11
Male, *n* (%)	488 (59.2)	110 (64.7)	378 (57.7)	0.11
**Body mas index, mean (SD)**	28.8 (4.9)	29.7 (4.8)	28.5 (5.0)	0.01
**Disease severity at the baseline, *n*** **(%)**				
Oxygen saturation 91–95%	466 (56.5)	58 (34.1)	408 (62.3)	<0.001
Oxygen saturation ≤90%	359 (43.5)	112 (65.9)	247 (37.7)	<0.001
Score on ordinal scale, *n* (%)				
4. Hospitalized, requiring no oxygen supplementation, but requiring medical care	163 (19.8)	11 (6.5)	152 (23.2)	<0.001
5. Hospitalized, requiring normal oxygen supplementation	615 (74.5)	147 (86.5)	468 (71.5)	<0.001
6. Hospitalized, on non-invasive ventilation with high-flow oxygen equipment	47 (5.7)	12 (7.1)	35 (5.3)	0.36
**Coexisting conditions, *n* (%)**	638 (77.3)	131 (77.1)	507 (77.4)	0.91
**Other medications related to COVID-19** **, *n*** **(%)**				
Remdesivir	284 (34.4)	67 (39.4)	217 (33.1)	0.15
Dexamethason	272 (33.0)	83 (48.8)	189 (28.9)	<0.001
Covalescent plasma	103 (12.5)	29 (17.1)	74 (11.3)	0.05
Low molecular weight heparin	713 (86.5)	170 (100.0)	543 (82.9)	<0.001

**Table 2:** Tocilizumab effect on rates of death, need for mechanical ventilation, and clinical improvement depending on possible outcomes predictors.

Outcomes	Tocilizumab	No Tocilizumab	*p*	OR (95%CI)
**Overall**				
*n*	170	655		
Death, *n* (%)	19 (11.2)	70 (10.7)	0.89	1.05 (0.61–1.80)
Mechanical ventillation, *n* (%)	11 (6.5)	39 (6.0)	0.86	1.09 (0.55–2.18)
Clinical improvement after 14 days, *n* (%)	70 (41.2)	351 (53.6)	**0.004**	**0.61** **(0.43–0.85)**
Clinical improvement after 21 days, *n* (%)	112 (65.9)	492 (75.1)	**0.02**	**0.64** **(0.44–0.92)**
Clinical improvement after 28 days, *n* (%)	134 (78.8)	531 (81.1)	0.51	0.87 (0.57–1.32)
**Age > 70 years**				
*n*	53	215		
Death, *n* (%)	11 (20.8)	49 (22.8)	0.85	0.89 (0.42–1.85)
Mechanical ventillation, *n* (%)	5 (9.4)	20 (9.3)	1.00	1.02 (0.36–2.84)
Clinical improvement after 14 days, *n* (%)	16 (30.2)	77 (35.8)	0.52	0.83 (0.43–1.59)
Clinical improvement after 21 days, *n* (%)	28 (52.8)	124 (57.7)	0.54	0.82 (0.45–1.50)
Clinical improvement after 28 days, *n* (%)	35 (66.0)	141 (65.6)	1.00	1.02 (0.54–1.92)
**The need for oxygen high flow (score 6 in ordinal scale) at baseline**				
*n*	14	35		
Death, *n* (%)	4 (28.6)	12 (34.3)	1.00	0.77 (0.20–2.97)
Mechanical ventillation, *n* (%)	4 (28.6)	8 (22.9)	0.72	1.35 (0.33–5.50)
Clinical improvement after 14 days, *n* (%)	6 (42.9)	9 (25.7)	0.31	2.17 (0.59–7.97)
Clinical improvement after 21 days, *n* (%)	9 (64.3)	15 (42.9)	0.21	2.40 (0.67–8.65)
Clinical improvement after 28 days, *n* (%)	9 (64.3)	17 (48.6)	0.36	1.91 (0.53–6.85)
**Clinical worsening during 7 days of hospitalization in patients with regular oxygen supplementation at baseline** **(5 points in original scale)**				
*n*	41	55		
Death, *n* (%)	18 (43.9)	37 (67.3)	**0.04**	**0.38** **(0.16–0.88)**
Mechanical ventillation, *n* (%)	20 (48.8)	23 (41.8)	0.54	1.32 (0.59–3.00)
Clinical improvement after 14 days, *n* (%)	0	2 (3.6)	0.51	-
Clinical improvement after 21 days, *n* (%)	1 (2.4)	8 (14.5)	0.07	0.15 (0.01–1.23)
Clinical improvement after 28 days, *n* (%)	14 (34.1)	12 (21.8)	0.24	1.85 (0.75–4.61)
**SpO2** **≤** **90% at the baseline**				
*n*	125	247		
Death, *n* (%)	23 (17.6)	52 (21.1)	0.59	0.84 (0.49–1.46)
Mechanical ventillation, *n* (%)	23 (17.6)	30 (11.7)	0.12	1.63 (0.90–2.95)
Clinical improvement after 14 days, *n* (%)	41 (32.8)	106 (42.5)	0.07	0.65 (0.41–1.10)
Clinical improvement after 21 days, *n* (%)	66 (52.8)	156 (62.8)	0.06	0.65 (0.42–1.01)
Clinical improvement after 28 days, *n* (%)	83 (66.4)	173 (69.6)	0.47	0.84 (0.53–1.34)
**IL-6 > 100 pg/mL** **at baseline**				
*n*	56	42		
Death, *n* (%)	6 (10.7)	13 (31.0)	**0.02**	**0.27** **(0.10–0.78)**
Mechanical ventillation, *n* (%)	7 (12.5)	7 (16.7)	0.57	0.71 (0.23–2.20)
Clinical improvement after 14 days, *n* (%)	22 (39.3)	14 (33.3)	0.67	1.29 (0.56–2.99)
Clinical improvement after 21 days, *n* (%)	35 (62.5)	19 (45.2)	0.10	2.02 (0.89–4.55)
Clinical improvement after 28 days, *n* (%)	41 (73.2)	23 (54.8)	0.08	2.26 (0.97–5.27)
**CRP > 200 mg/L** **at the baseline**				
*n*	39	61		
Death, *n* (%)	6 (15.4)	13 (21.3)	0.60	0.74 (0.25–2.17)
Mechanical ventillation, *n* (%)	6 (15.4)	10 (16.4)	1.00	0.92 (0.31–2.79)
Clinical improvement after 14 days, *n* (%)	12 (30.8)	17 (27.9)	0.82	1.15 (0.48–2.78)
Clinical improvement after 21 days, *n* (%)	20 (51.3)	35 (57.4)	0.68	0.78 (0.35–1.75)
Clinical improvement after 28 days, *n* (%)	27 (69.2)	39 (63.9)	0.67	1.27 (0.54–2.99)
**Neutrophils > 7500/µL** **at the baseline**				
*n*	39	90		
Death, *n* (%)	7 (17.9)	23 (25.6)	0.49	0.63 (0.25–1.64)
Mechanical ventillation, *n* (%)	2 (5.1)	8 (8.9)	1.00	0.55 (0.11–2.73)
Clinical improvement after 14 days, *n* (%)	15 (38.5)	33 (36.7)	0.84	1.08 (0.50–2.34)
Clinical improvement after 21 days, *n* (%)	23 (59.0)	51 (56.7)	0.84	1.10 (0.51–2.35)
Clinical improvement after 28 days, *n* (%)	28 (71.8)	59 (65.6)	0.54	1.34 (0.59–3.04)
**Lymphocytes > 1200/µL** **at the baseline**				
*n*	47	239		
Death, *n* (%)	1 (2.1)	20 (8.4)	0.22	0.24 (0.03–1.82)
Mechanical ventillation, *n* (%)	0	8 (3.3)	0.36	-
Clinical improvement after 14 days, *n* (%)	28 (59.6)	142 (59.4)	1.00	1.01 (0.53–1.90)
Clinical improvement after 21 days, *n* (%)	36 (76.6)	192 (80.3)	0.55	0.80 (0.38–1.69)
Clinical improvement after 28 days, *n* (%)	43 (91.5)	201 (84.1)	0.26	2.03 (0.69–6.00)
**D-dimers > 1000 µg/L** **at the baseline**				
*n*	75	221		
Death, *n* (%)	12 (16.0)	45 (20.4)	0.50	0.74 (0.37–1.50)
Mechanical ventillation, *n* (%)	7 (9.3)	21 (9.5)	1.00	0.98 (0.40–2.41)
Clinical improvement after 14 days, *n* (%)	25 (33.3)	101 (45.7)	0.08	0.59 (0.34–1.03)
Clinical improvement after 21 days, *n* (%)	43 (57.3)	143 (64.7)	0.27	0.73 (0.43–1.25)
Clinical improvement after 28 days, *n* (%)	54 (72.0)	158 (71.5)	1.00	1.02 (0.57–1.84)
**Procalcitonin > 0.1 ng/mL** **at the baseline**				
*n*	92	193		
Death, *n* (%)	18 (19.6)	44 (22.8)	0.64	0.82 (0.44–1.52)
Mechanical ventillation, *n* (%)	10 (10.9)	25 (13.0)	0.70	0.82 (0.37–1.79)
Clinical improvement after 14 days, *n* (%)	34 (37.0)	74 (38.3)	0.89	0.94 (0.56–1.57)
Clinical improvement after 21 days, *n* (%)	53 (57.6)	117 (60.6)	0.70	0.88 (0.53–1.46)
Clinical improvement after 28 days, *n* (%)	68 (73.9)	128 (66.3)	0.22	1.44 (0.83–2.50)

Bold: statistical significance.

**Table 3:** Correlations between baseline serum IL-6 vs. selected clinical and laboratory indices.

IL-6 Versus	All Patients (*n* = 825)		SpO2 ≤ 90% (*n* = 372)		SpO2 91–95% (*n* = 453)	
	**r_s_**	***p***	**r_s_**	***p***	**r_s_**	***p***
**Age**	0.15	<0.001	0.07	0.32	0.18	0.002
**BMI**	0.01	0.86	−0.03	0.63	0.01	0.89
**SpO_2_**	−0.33	<0.001	−0.19	0.003	−0.31	<0.001
**CRP**	0.58	<0.001	0.44	<0.001	0.68	<0.001
**Procalcitonin**	0.40	<0.001	0.37	<0.001	0.36	<0.001
**WBC**	0.26	<0.001	0.22	<0.001	0.25	<0.001
**Lymphocytes**	−0.21	<0.001	−0.05	0.42	−0.28	<0.001
**Neutrophils**	0.32	<0.001	0.23	<0.001	0.37	<0.001
**Platelets**	−0.10	0.04	−0.10	0.14	−0.13	0.03
**D-dimers**	0.28	<0.001	0.20	0.003	0.27	<0.001
**ALT**	0.13	0.03	0.10	0.13	0.10	0.09

**Table 4:** Tocilizumab effect on rates of death, need for mechanical ventilation, and clinical improvement depending on combinations of possible outcomes predictors.

Outcomes	Tocilizumab	No Tocilizumab	*p*	OR (95%CI)
**Baseline IL6 > 100 pg/mL and requiring normal or high-flow oxygen supplementation (5 or 6 scores in ordinal scale)**				
***n***	53	34		
Death, *n* (%)	8 (15.1)	13 (38.2)	**0.02**	**0.18** **(0.06–0.52)**
Mechanical ventillation, *n* (%)	8 (15.1)	7 (20.6)	0.57	0.68 (0.22–2.10)
Clinical improvement after 14 days, *n* (%)	20 (37.7)	10 (29.4)	0.49	1.45 (0.58–3.66)
Clinical improvement after 21 days, *n* (%)	33 (62.3)	13 (38.2)	**0.047**	**2.66** **(1.10–6.47)**
Clinical improvement after 28 days, *n* (%)	38 (71.7)	16 (47.1)	**0.02**	**2.85** **(1.16–7.01)**
**Baseline IL6 > 100 pg/mL and SpO2 < 90%**				
***n***	37	24		
Death, *n* (%)	4 (10.8)	12 (50.0)	**<0.001**	**0.07** **(0.02–0.27)**
Mechanical ventillation, *n* (%)	2 (5.4)	7 (29.2)	**0.02**	**0.14** **(0.03–0.74)**
Clinical improvement after 14 days, *n* (%)	12 (32.4)	3 (12.5)	0.12	3.36 (0.83–13.52)
Clinical improvement after 21 days, *n* (%)	24 (64.9)	6 (25.0)	**0.004**	**5.53** **(1.76–17.40)**
Clinical improvement after 28 days, *n* (%)	28 (75.7)	9 (37.5)	**0.004**	**5.18** **(1.70–17.84)**
**Baseline IL6 > 100 pg/mL and CRP > 200 mg/L**				
***n***	32	26		
Death, *n* (%)	5 (15.6)	7 (26.9)	0.34	0.50 (0.14–1.82)
Mechanical ventillation, *n* (%)	1 (3.1)	4 (15.4)	0.16	0.18 (0.02–1.70)
Clinical improvement after 14 days, *n* (%)	11 (34.4)	8 (30.8)	1.00	1.18 (0.39–3.57)
Clinical improvement after 21 days, *n* (%)	18 (56.3)	15 (57.7)	1.00	0.94 (0.33–2.68)
Clinical improvement after 28 days, *n* (%)	24 (75.0)	18 (69.2)	0.77	1.33 (0.42–4.23)
**Baseline IL6 > 100 pg/mL and CRP > 200 mg/L and SpO2 < 90%**				
***n***	21	13		
Death, *n* (%)	4 (19.0)	5 (38.5)	0.26	0.37 (0.08–1.80)
Mechanical ventillation, *n* (%)	1 (4.8)	3 (23.1)	0.27	0.17 (0.01–1.81)
Clinical improvement after 14 days, *n* (%)	6 (28.6)	3 (23.1)	1.00	1.33 (0.27–6.61)
Clinical improvement after 21 days, *n* (%)	11 (52.4)	5 (38.5)	0.49	1.76 (0.43–7.19)
Clinical improvement after 28 days, *n* (%)	15 (71.4)	8 (61.5)	0.71	1.56 (0.36–6.76)

**Table 5:** Prevalence of adverse events.

Adverse Events	Tocilizumab	No Tocilizumab	*p*
***n***	170	655	
ALT elevation, *n* (%)	17 (10.0)	40 (6.1)	0.09
Diarrhea, *n* (%)	8 (4.7)	39 (5.9)	0.71
Prolonged QT interval, *n* (%)	3 (1.8)	11 (1.7)	1.00
Nausea, *n* (%)	2 (1.2)	12 (1.8)	0.75
Other, *n* (%)	7 (4.7)	11 (1.7)	0.07
All adverse events, *n* (%)	37 (21.7)	113 (17.3)	0.18

## Data Availability

Data supporting reported results can be provided upon request from the corresponding author.
